# Genetic interaction between DNA repair factors *PAXX*, *XLF, XRCC4* and *DNA‐PKcs* in human cells

**DOI:** 10.1002/2211-5463.12681

**Published:** 2019-06-12

**Authors:** Mengtan Xing, Valentyn Oksenych

**Affiliations:** ^1^ Department of Clinical and Molecular Medicine (IKOM) Norwegian University of Science and Technology Trondheim Norway

**Keywords:** chromosomal breaks, DNA damage response, double‐strand breaks, genetic interaction, HAP1, NHEJ

## Abstract

DNA double‐strand breaks (DSBs) are highly cytotoxic lesions, and unrepaired or misrepaired DSBs can lead to various human diseases, including immunodeficiency, neurological abnormalities, growth retardation, and cancer. Nonhomologous end joining (NHEJ) is the major DSB repair pathway in mammals. Ku70 and Ku80 are DSB sensors that facilitate the recruitment of downstream factors, including protein kinase DNA‐dependent protein kinase, catalytic subunit (DNA‐PKcs), structural components [X‐ray repair cross‐complementing protein 4 (XRCC4), XRCC4‐like factor (XLF), and paralogue of XRCC4 and XLF (PAXX)], and DNA ligase IV (LIG4), which complete DNA repair. DSBs also trigger the activation of the DNA damage response pathway, in which protein kinase ataxia‐telangiectasia mutated (ATM) phosphorylates multiple substrates, including histone H2AX. Traditionally, research on NHEJ factors was performed using *in vivo* mouse models and murine cells. However, the current knowledge of the genetic interactions between NHEJ factors in human cells is incomplete. Here, we obtained genetically modified human HAP1 cell lines, which lacked one or two NHEJ factors, including LIG4, XRCC4, XLF, PAXX, DNA‐PKcs, DNA‐PKcs/XRCC4, and DNA‐PKcs/PAXX. We examined the genomic instability of HAP1 cells, as well as their sensitivity to DSB‐inducing agents. In addition, we determined the genetic interaction between XRCC4 paralogues (XRCC4, XLF, and PAXX) and DNA‐PKcs. We found that in human cells, XLF, but not PAXX or XRCC4, genetically interacts with DNA‐PKcs. Moreover, ATM possesses overlapping functions with DNA‐PKcs, XLF, and XRCC4, but not with PAXX in response to DSBs. Finally, NHEJ‐deficient HAP1 cells show increased chromosomal and chromatid breaks, when compared to the WT parental control. Overall, we found that HAP1 is a suitable model to study the genetic interactions in human cells.

**Abbreviations**
ATM: ataxia‐telangiectasia mutated
DNA‐PKcs: DNA‐dependent protein kinase, catalytic subunit
DSBs: DNA double‐strand breaks
HAP1: a near‐haploid human cell line derived from KBM‐7 cell line
HR: homologous recombination
Ku: Ku70/Ku80 heterodimer
LIG4: DNA ligase IV
MRI: modulator of retroviral infection
MRN: Mre11/Rad50/Nbs1
NHEJ: nonhomologous end joining
PAXX: paralogue of XRCC4 and XLF
T‐FISH: telomere fluorescence *in* *situ* hybridization
WB: western blot
XLF: XRCC4‐like factor
XRCC4: X‐ray repair cross‐complementing protein 4

DNA double‐strand break (DSB) is the most deleterious type of DNA lesion to a cell, as unrepaired breaks can be lethal to a cell, and incorrect repair can cause gross genetic rearrangements 1, 2, 3. For research purposes, DSBs are often induced by exogenous sources, for instance, ionizing radiation and chemotherapeutic drugs 4. In developing lymphocytes, programmed DSBs are generated during physiological processes, such as V(D)J recombination and immunoglobulin heavy chain class switch recombination 1, 3.

In mammalian cells, there are two major DSB repair pathways: homologous recombination (HR) and nonhomologous DNA end joining (NHEJ) 2. HR is dependent on sister chromatids as templates, and it is restricted to the S/G2 phases of the cell cycle 5. Unlike HR, NHEJ can be active throughout the cell cycle 6. NHEJ consists of core and accessory factors. The core factors consist of Ku70, Ku80, X‐ray repair cross‐complementing protein 4 (XRCC4), and DNA ligase IV (LIG4), while the accessory factors include XRCC4‐like factor (XLF), DNA‐dependent protein kinase catalytic subunit (DNA‐PKcs), paralogue of XRCC4 and XLF (PAXX), and modulator of retroviral infection (MRI). During decades, the vast majority of the cutting‐edge research on NHEJ and the accumulated knowledge on the role of individual NHEJ factors was based on traditionally single loss‐of‐function cellular and *in vivo* mouse models. The paradigm stated that the core NHEJ factors are required for DSB repair in the absence of HR, while the accessory NHEJ factors are dispensable for both robust DNA repair and mouse development 7, 8. However, during the last several years, it became clear that accessory NHEJ factors indeed are required for efficient DNA repair, although their function is less obvious due to complex genetic interactions between, for instance, XLF and DNA‐PKcs 9, XLF and PAXX 10, 11, 12, 13, and XLF and MRI 8.

Epistasis is a kind of genetic interactions, and it is operationally defined by the use of mutant strains. If the presence of mutations in two different genetic loci confers a phenotype (e.g. sensitivity to UV radiation), which is quantitatively the same as that conferred by every single mutation alone, the two genes are said to be epistatic to one another. In contrast, if mutations in two different genes confer additive effects (e.g. increased UV radiation sensitivity), they are placed in different epistasis groups 14. Additionally, when two or more genes, proteins, or pathways perform similar, interchangeable activities, they are defined as functionally redundant 15.

Synthetic lethality occurs between genes with redundant functions. For example, DNA‐PKcs is a serine/threonine kinase, a member of the phosphatidylinositol‐3‐kinase‐like kinase family, which also includes ataxia‐telangiectasia mutated (ATM) protein kinase 16. A homozygous mutation in the murine *Dna‐pkcs* leading to a C‐terminal truncation of the protein results in severe combined immunodeficiency, SCID 17, and a kinase‐dead DNA‐PKcs‐mutated protein leads to Ku70/Ku80‐ and p53‐dependent embryonic lethality in mice 18. While *Dna‐pkcs *(*Prkdc*) and *Atm* single‐knockout mice are viable, *Dna‐pkcs*/*Atm* double‐knockout mice are embryonically lethal 19, 20, 21. Thus, DNA‐PKcs is functionally redundant with ATM in mice 21, 22. However, no or limited information on such genetic interaction in human cells is available.

To elucidate the genetic interactions between DNA‐PKcs and the XRCC4 paralogues (XRCC4, XLF, PAXX) in human cells, we used knockout human HAP1 cell lines 23. HAP1 is a nearly haploid cell line, and it is a suitable model being used to address the impact of gene functions 24, 25, 26.

Here, we obtained *XRCC4^∆^*‐*, XLF^∆^*‐*, PAXX^∆^*‐*, DNA‐PKcs^∆^*‐*, DNA‐PKcs^∆^ PAXX^∆^*‐*, DNA‐PKcs^∆^ XRCC4^∆^*‐*, LIG4^∆^*‐, and *H2AX^∆^*‐knockout HAP1 cell lines, all derived from the parental HAP1 cell line. By exposing these cells to the DNA‐PKcs kinase inhibitor NU7441, DSB‐inducing reagent etoposide (Eto), and measuring the genomic stability in these cells, we found that DNA‐PKcs functions redundantly with XLF but epistatically with XRCC4 in Eto resistance. Moreover, we exposed the HAP1 cells to ATM kinase inhibitor KU55933 and found that ATM functions redundantly with DNA‐PKcs, XLF, and XRCC4 in Eto resistance.

## Materials and methods

### Chemicals

NU7441 (Selleckchem, Houston, TX, USA; S2638), Eto (Sigma‐Aldrich, St. Louis, MO, USA; E1383), KU55933 (Sigma, SML1109), PrestoBlue^TM^ Cell Viability Reagent (Thermo Fisher, Waltham, MA, USA; A13262), KaryoMAX^TM^ Colcemid^TM^ Solution in PBS (Thermo Fisher, 15212012), TelC‐Cy3 (Panagene, Daejeon, South Korea, F1002‐5), and VECTASHIELD Antifade Mounting Medium with DAPI (Vector Laboratories, Burlingame, CA, USA; H‐1200) were used.

### Cell culture

All the NHEJ‐deficient HAP1 cell lines are derived from the parental HAP1 cell line. They are nearly haploid cells and were custom‐generated by request and provided by Horizon Discovery. HAP1 cells were cultured in Iscove's Modified Dulbecco's Medium (IMDM; Thermo Fisher, 21980065) supplemented with 10% FBS (Sigma, F7524), and 1% Penicillin‐Streptomycin (Thermo Fisher, 15140122) at 37 °C with 5% CO_2_, according to the manufacturer's instructions.

### Antibodies

Antibodies used for western blot (WB) include rabbit polyclonal anti‐PAXX (C9orf142; 1 : 1000 dilution; Novus Biologicals, Centennial, CO, USA; NBP1‐94172), anti‐XLF (1 : 1000; Cell Signaling, 2854), anti‐LIG4 (1 : 1000; Abcam, Cambridge, UK, ab193353), anti‐H2AX (1 : 5000; Abcam, ab11175); mouse monoclonal anti‐DNA‐PKcs (1 : 1000; Invitrogen, MA5‐13404), anti‐XRCC4 (1 : 2000; Novus Biologicals, NBP1‐48053), anti‐β‐actin (1 : 2000; Abcam, ab8226); swine polyclonal anti‐rabbit Ig‐HRP (1 : 5000; Dako, Santa Clara, CA, USA; P0399); goat polyclonal anti‐mouse Ig‐HRP (1 : 5000; Dako, P0447); IRDye® 800CW Goat anti‐Rabbit IgG (H + L; LI‐COR, P/N 926‐32211); and IRDye® 680RD Goat anti‐Mouse IgG (H + L; LI‐COR, P/N 926‐68070).

### Cell survival assay

The sensitivity of HAP1 cells to DSB‐inducing agent was measured using the PrestoBlue (Thermo Fisher Scientific, Waltham, MA, USA) assay according to the manufacturer's instructions. The cells were seeded into 96‐well plates at a cell density of 2000–5000 per well in 100 µL IMDM; 24 h later, 50 µL of media was removed and 50 µL of media containing, when indicated, Eto, NU7441 or KU55933, was added to the cells. After 72 h, 11 µL of PrestoBlue (10×) cell viability reagent was added to the cells, followed by 30 min of incubation at 37 °C. Cell viability was measured with a fluorescence multi‐well reader with the excitation/emission wavelengths set at 544/590 nm, and each dose point was measured in triplicates.

### T‐FISH

HAP1 cells were incubated at 37 °C in IMDM with 0.1 μg·mL^−1^ colcemid for 5 h. Afterward, the media was collected, and the cells were trypsinized and then combined with the media collected earlier. Next, the cells were lysed in the hypotonic solution (75 mm KCl) for 15 min at 37 °C, subsequently fixed in methanol: acetic acid (3 : 1) solution, and air‐dried on slides overnight. After digestion with pepsin (1 mg·mL^−1^, 10 min at 37 °C), the slides were heated at 80 °C for 3 min to denature DNA, which was then hybridized with a Cy3‐labeled PNA FISH probe (TelC‐Cy3) in 70% formamide at room temperature for 2 h, washed, dehydrated, and mounted in VECTASHIELD Antifade Mounting Medium with DAPI, as described previously 9, 26, 27, 28, 29. Metaphase images were captured using a Zeiss TRIF 3 microscope (Oberkochen, Germany) equipped with CCD cameras and a 100× objective lens [Cellular and Molecular Imaging Core (CMIC) facility, NTNU, Trondheim, Norway].

### Colony formation assay

One hundred HAP1 cells were seeded in each well of six‐well plates in 2 mL IMDM and treated with Eto at indicated concentrations in triplicates. At day 14, the colonies were stained with crystal violet (0.5%) and counted, as described previously 26, 30, 31.

## Results

### 1 μm NU7441 inhibit specifically DNA‐PKcs kinase

**Figure 1:**
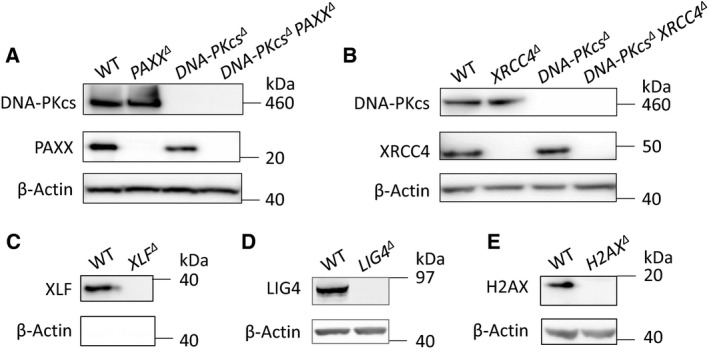
Verification of HAP1 cells. WB analyses of DNA‐PKcs and PAXX expression in WT, *PAXX^∆^*, *DNA‐PKcs^∆^*, and *DNA‐PKcs^∆^ PAXX^∆^* HAP1 cells (A); expression of DNA‐PKcs and XRCC4 in WT, *XRCC4^∆^*, *DNA‐PKcs^∆^ XRCC4^∆^* HAP1 cells (B); expression of XLF (C), LIG4 (D) and H2AX (E) in WT, *XLF^∆^*, *LIG4^∆^*, and *H2AX^∆^* HAP1 cells; β‐actin was used as a loading control for WB.

**Figure 2:**
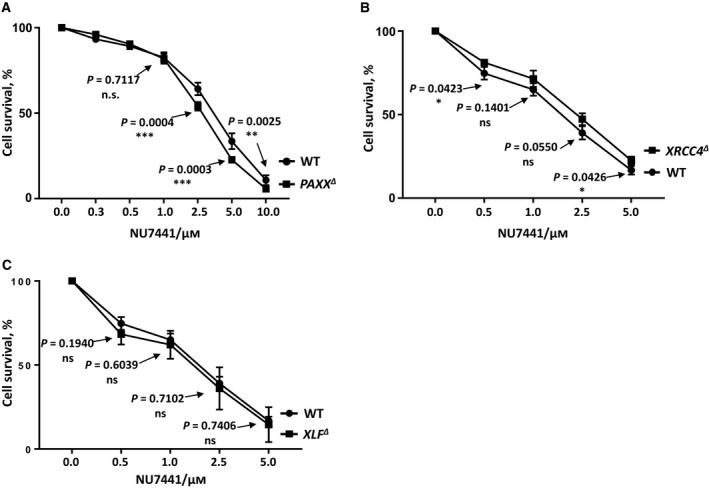
DNA‐PKcs inhibitor NU7441 toxicity test in HAP1 cells. Cell survival assay was performed in *PAXX^∆^* (A), *XRCC4^∆^* (B), *XLF^∆^* (C), and WT HAP1 cell lines to test the toxicity of DNA‐PKcs inhibitor NU7441. Results are from the mean (SD) of three independent experiments. The labels are in according to the cell line sensitivity severity from up to bottom. The comparisons between WT and the knockout cell lines at each dose point were obtained with unpaired *t*‐test using graphpad prism 7.03 (San Diego, CA, USA), and the *P* values were indicated in the graphs. *P* < 0.05 (*); *P* < 0.01 (**); *P* < 0.001 (***).

To determine the genetic interaction between DNA‐PKcs and the XRCC4 paralogues, we obtained multiple NHEJ‐knockout human HAP1 cell lines (Table 1 and Fig. 1). First, we exposed *PAXX^∆^* (Fig. 2A), *XRCC4^∆^* (Fig. 2B), *XLF^∆^* (Fig. 2C), and WT HAP1 cells to DNA‐PKcs inhibitor NU7441 and found that the sensitivity of *PAXX^∆^*, *XRCC4^∆^*, *XLF^∆^*, and WT HAP1 cells to NU7441 remained the same at 1 μm.

**Table 1:** CRISPR/Cas9‐induced mutagenesis of the NHEJ genes and gene locations in Homo sapiens.

Cell line	CRISPR/Cas9 target mutant (Horizon Discovery)	Catalog number (Horizon Discovery)	Cytogenetic location (OMIM)
*PAXX^∆^*	1bp insertion in exon 4	HZGHC004376c005	9q34.3
*DNA‐PKcs^∆^*	11bp deletion in exon 25	HZGHC024034c011	8q11.21
*DNA‐PKcs^∆^ PAXX^∆^*	11bp deletion in exon 25 for DNA‐PKcs 28bp deletion in exon 4 for PAXX	HZGHC005239c011	
*XRCC4^∆^*	8bp deletion in exon 2	HZGHC000428c019	5q14.2
*DNA‐PKcs^∆^ XRCC4^∆^*	11bp deletion in exon 25 for DNA‐PKcs 1bp insertion in exon 2 for XRCC4	HZGHC005633c001	
*XLF^∆^*	4bp deletion in exon 3	HZGHC000426c017	2q35
*LIG4^∆^*	10bp deletion in exon 2	HZGHC000759c005	13q33.3
*H2AX^∆ar^*	2bp deletion in exon 1	HZGHC005630c003	11q23.3

**Figure 3:**
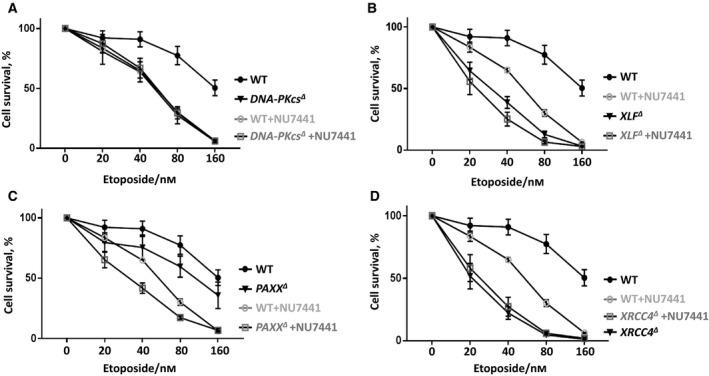
DNA‐PKcs functions redundantly with XLF. Sensitization of HAP1 WT and *DNA‐PKcs^∆^* (A), *XLF^∆^* (B), *PAXX^∆^* (C), *XRCC4^∆^* (D) cells to Eto with or without 1 μm NU7441 treatment. Results are from the mean (SD) of three independent experiments. The labels are in according to the cell line sensitivity severity from up to bottom. Comparisons between each two groups at 40 nm Eto were obtained with unpaired *t*‐test using graphpad prism 7.03: WT + NU7441 (65%) vs WT (91%), *P* = 0.0023 (**); *DNA‐PKcs^∆^* (64%) vs WT (91%), *P* = 0.0108 (*); *DNA‐PKcs^∆^ *+ NU7441 (67%) vs WT + NU7441 (65%), *P* = 0.6991 (n.s.); *DNA‐PKcs^∆^ *+ NU7441 (67%) vs* DNA‐PKcs^∆^* (64%), *P* = 0.6634 (n.s.). *XLF^∆^* (39%) vs WT (91%), *P* = 0.0003 (***); *XLF^∆^ *+ NU7441 (25%) vs WT + NU7441 (65%), *P* = 0.0003 (***); *XLF^∆^ *+ NU7441 (25%) vs* XLF^∆^* (39%), *P* = 0.0342 (*). *PAXX^∆^* (76%) vs WT (91%), *P* = 0.0898 (n.s.); *PAXX^∆^ *+ NU7441 (42%) vs WT + NU7441 (65%), *P* = 0.0010 (***); *PAXX^∆^ *+ NU7441 (42%) vs* PAXX^∆^* (76%), *P* = 0.0062 (**); *XRCC4^∆^* (22%) vs WT (91%), *P* = 0.0001 (***); *XRCC4^∆^ *+ NU7441 (27%) vs WT + NU7441 (65%), *P* = 0.0011 (**); *XRCC4^∆^ *+ NU7441 (27%) vs* XRCC4^∆^* (22%), *P* = 0.3934 (n.s.).

In addition, we ensured the specificity of DNA‐PKcs inhibitor NU7441 using DNA‐PKcs‐knockout HAP1 cell line. We found that WT cells exposed to 1 μm NU7441 possessed similar sensitivity to Eto as *DNA‐PKcs^∆^* HAP1 cells (Fig. 3A). Of note, we observed no additional effect of NU7441 to *DNA‐PKcs^∆^* HAP1 cells on Eto sensitivity (Fig. 3A). Thus, for further experiments, we used 1 μm NU7441 to inhibit specifically DNA‐PKcs kinase.

### DNA‐PKcs functions redundantly with XLF in HAP1 cells

DNA‐PKcs is functionally redundant with XLF in mouse development and DNA repair 9, 28, 32. To determine how *DNA‐PKcs* and *XLF* interact genetically in human cells, we exposed *XLF^∆^* and WT HAP1 cells to 1 μm of DNA‐PKcs inhibitor NU7441 combined with Eto and observed that *XLF^∆^* cells treated with NU7441 possessed modest although significant hypersensitivity to Eto (40 nm) when compared to WT and *XLF^∆^* controls (*P* < 0.0343*, Fig. 3B). Thus, we concluded that DNA‐PKcs and XLF have redundant functions during Eto‐induced DSB response in HAP1 cells.

### Genetic interaction between *PAXX, XRCC4, and DNA‐PKcs*

To determine the possible genetic interaction between *PAXX*, *XRCC4,* and *DNA‐PKcs*, we exposed *PAXX^∆^* (Fig. 3C), *XRCC4^∆^* (Fig. 3D), and WT HAP1 cells to DNA‐PKcs inhibitor NU7441 combined with Eto, as described above. We observed that the inhibition of DNA‐PKcs in *PAXX^∆^* cells resulted in mild hypersensitivity to Eto (40 nm) when compared to WT controls (*P* = 0.0010***; Fig. 3C), while there was no significant change of the sensitivity to Eto (40 nm) in *XRCC4^∆^* HAP1 cells (*P* = 0.3934, Fig. 3D).

**Figure 4:**
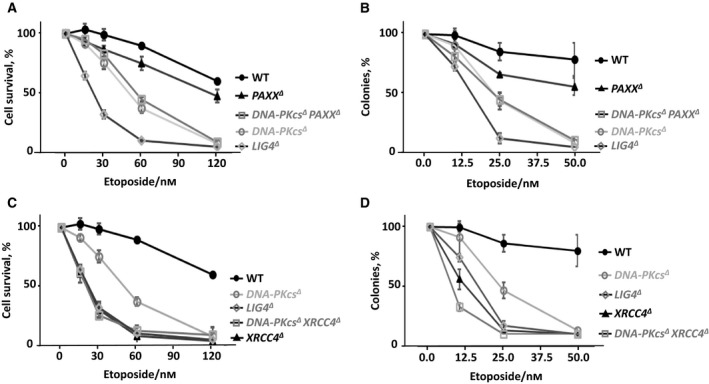
DNA‐PKcs functions epistatically with PAXX and XRCC4. (A) The sensitivity of WT, *PAXX^∆^*, *DNA‐PKcs^∆^, DNA‐PKcs^∆^ PAXX^∆^*, and *LIG4^∆^* HAP1 cells to Eto. Results are from the mean (SD) of three independent experiments. The *y*‐axis (cell survival %) is the relative percentage of the fluorescence‐based nucleotide dye to untreated cells. (B) Colony formation assay of WT, *PAXX^∆^*, *DNA‐PKcs^∆^, DNA‐PKcs^∆^ PAXX^∆^*, and *LIG4^∆^* HAP1 cells to Eto. Results are from the mean (SD) of three independent experiments. The *y*‐axis (colonies) is the relative number of colonies to untreated cells. (C) WT, *XRCC4^∆^*, *DNA‐PKcs^∆^, DNA‐PKcs^∆^ XRCC4^∆^*, and *LIG4^∆^* HAP1 cells to Eto. Results are from the mean (SD) of three independent experiments. The *y*‐axis (cell survival %) is the relative percentage of the fluorescence‐based nucleotide dye to untreated cells. (D) Colony formation assay of WT, *XRCC4^∆^*, *DNA‐PKcs^∆^, DNA‐PKcs^∆^ XRCC4^∆^*, and *LIG4^∆^* HAP1 cells to Eto. Results are from the mean (SD) of three independent experiments. The *y*‐axis (colonies) is the relative number of colonies to untreated cells. *LIG4^∆^* HAP1 cell line was used as negative control. The labels are in according to the cell line sensitivity severity from up to bottom. Comparisons between each two groups were obtained with ordinary one‐way ANOVA using graphpad prism 7.03. For (A) and (C) at 64 nm Eto: WT (90%) vs* PAXX^∆^* (74%), *P* = 0.0594 (n.s.); WT (90%) vs* DNA‐PKcs^∆^* (36%), *P* < 0.0001 (****); WT (90%) vs* DNA‐PKcs^∆^ PAXX^∆^* (43%), *P* < 0.0001 (****); WT (90%) vs* LIG4^∆^* (8%), *P* < 0.0001 (****); *PAXX^∆^* (74%) vs* DNA‐PKcs^∆^* (36%), *P* = 0.0001 (***); *PAXX^∆^* (74%) vs* DNA‐PKcs^∆^ PAXX^∆^* (43%), *P* = 0.0007 (***); *PAXX^∆^* (74%) vs* LIG4^∆^* (8%), *P* < 0.0001 (****); *DNA‐PKcs^∆^* (36%) vs* DNA‐PKcs^∆^ PAXX^∆^* (43%), *P* = 0.4245 (n.s.); *DNA‐PKcs^∆^* (36%) vs* LIG4^∆^* (8%), *P* = 0.0007 (***); *DNA‐PKcs^∆^ PAXX^∆^* (43%) vs* LIG4^∆^* (8%), *P* = 0.0001 (***). WT (90%) vs* XRCC4^∆^* (6%), *P* < 0.0001 (****); WT (90%) vs* DNA‐PKcs^∆^ XRCC4^∆^* (10%), *P* < 0.0001 (****); *XRCC4^∆^* (6%) vs* DNA‐PKcs^∆^* (36%), *P* < 0.0001 (****); *XRCC4^∆^* (6%) vs* DNA‐PKcs^∆^ XRCC4^∆^* (10%), *P* = 0.3571 (n.s.); *XRCC4^∆^* (6%) vs* LIG4^∆^* (8%), *P* = 0.8968 (n.s.); *DNA‐PKcs^∆^* (36%) vs* DNA‐PKcs^∆^ XRCC4^∆^* (10%), *P* < 0.0001 (****); *DNA‐PKcs^∆^ XRCC4^∆^* (10%) vs* LIG4^∆^* (8%), *P* = 0.8230 (n.s.). For (B) and (D)at 25 nm Eto: WT (85%) vs* PAXX^∆^* (64%), *P* = 0.0350 (*); WT (85%) vs* DNA‐PKcs^∆^* (41%), *P* < 0.0001 (****); WT (85%) vs* DNA‐PKcs^∆^ PAXX^∆^* (42%), *P* = 0.0001 (***); WT (85%) vs* LIG4^∆^* (8%), *P* < 0.0001 (****); *PAXX^∆^* (64%) vs* DNA‐PKcs^∆^* (41%), *P* = 0.0153 (*); *PAXX^∆^* (64%) vs* DNA‐PKcs^∆^ PAXX^∆^* (42%), *P* = 0.0209 (*); *PAXX^∆^* (64%) vs* LIG4^∆^* (8%), *P* < 0.0001 (****); *DNA‐PKcs^∆^* (41%) vs* DNA‐PKcs^∆^ PAXX^∆^* (42%), *P* = 0.9990 (n.s.); *DNA‐PKcs^∆^* (41%) vs* LIG4^∆^* (8%), *P* = 0.0007 (***); *DNA‐PKcs^∆^ PAXX^∆^* (42%) vs* LIG4^∆^* (8%), *P* = 0.0005 (***). WT (85%) vs* XRCC4^∆^* (4%), *P* < 0.0001 (****); WT (85%) vs* DNA‐PKcs^∆^ XRCC4^∆^* (0%), *P* < 0.0001 (****); *XRCC4^∆^* (4%) vs* DNA‐PKcs^∆^* (41%), *P* < 0.0001 (****); *XRCC4^∆^* (4%) vs* DNA‐PKcs^∆^ XRCC4^∆^* (0%), *P* = 0.9021 (n.s.); *XRCC4^∆^* (4%) vs* LIG4^∆^* (8%), *P* = 0.8658 (n.s.); *DNA‐PKcs^∆^* (41%) vs* DNA‐PKcs^∆^ XRCC4^∆^* (0%), *P* < 0.0001 (****); *DNA‐PKcs^∆^ XRCC4^∆^* (0%) vs* LIG4^∆^* (8%), *P* = 0.4088 (n.s.).

The effects described above might depend on the DNA‐PKcs catalytic activity or its physical presence. While exposing the HAP1 cells to 1 μm NU7441 is a model to study enzymatic activity, genetic inactivation (knockout) allows to determine the impact of DNA‐PKcs physical presence on the DNA repair in HAP1 cells. We obtained *DNA‐PKcs^∆^ PAXX^∆^* and *DNA‐PKcs^∆^ XRCC4^∆^* double‐knockout cell lines and exposed these cells to Eto. We observed that *DNA‐PKcs^∆^ PAXX^∆^* HAP1 cells possessed similar sensitivity to Eto as *DNA‐PKcs^∆^* HAP1 cells (Fig. 4A,B). While *LIG4^∆^* HAP1 cells exhibited stronger sensitivity to Eto than *DNA‐PKcs^∆^* HAP1 cells (Fig. 4A,B), *XRCC4^∆^* and *LIG4^∆^* HAP1 cell lines had similar sensitivity to Eto (Fig. 4c,D), and *DNA‐PKcs^∆^ XRCC4^∆^* HAP1 cells possessed similar sensitivity to Eto as *XRCC4^∆^* HAP1 cells (Fig. 4C,D). We concluded that DNA‐PKcs functions epistatically with PAXX and XRCC4 in Eto resistance in human HAP1 cells, and we proposed an explanation for the difference between the results obtained using *DNA‐PKcs^∆^ PAXX^∆^* double‐knockout cells and *PAXX^∆^* cells treated with DNA‐PKcs inhibitor NU7441 in the Discussion part.

### Genetic interactions between *ATM* and the *XRCC4* paralogues in HAP1 cells

**Figure 5:**
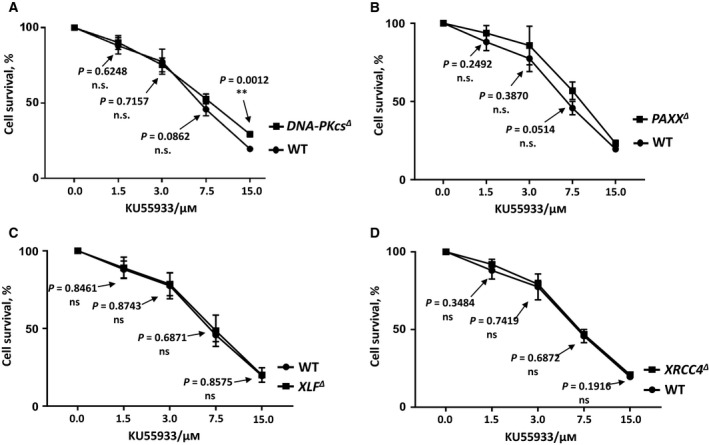
WT and NHEJ‐knockout HAP1 cell lines have similar sensitivity to ATM inhibitor KU55933. The sensitivity of WT and *DNA‐PKcs^∆^* (A), *PAXX^∆^* (B), *XLF^∆^* (C), *XRCC4^∆^* (D) HAP1 cells to ATM inhibitor KU55933. Results are from the mean (SD) of three independent experiments. The *y*‐axis (cell survival %) is the relative percentage of the fluorescence‐based nucleotide dye to untreated cells. The labels are in according to the cell line sensitivity severity from up to bottom. The comparisons between WT and the knockout cell lines at each dose point were obtained with unpaired *t*‐test using graphpad prism 7.03, and the *P* values were indicated in the graphs. WT + KU55933 vs DNA‐PKcs + KU55933, *P* < 0.0012 (**).

**Figure 6:**
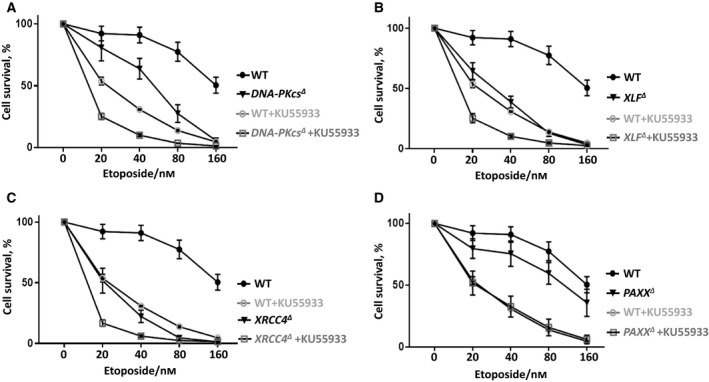
ATM functions redundantly with DNA‐PKcs, XLF, XRCC4, but not PAXX in Eto resistance. Sensitivity of WT and *DNA‐PKcs^∆^* (A), *XLF^∆^* (B), *XRCC4^∆^* (C), *PAXX^∆^* (D) HAP1 cells to Eto and 7.5 μm of KU55933. Results are from the mean (SD) of three independent experiments. The *y*‐axis (cell survival %) is the relative percentage of the fluorescence‐based nucleotide dye to untreated cells. The labels are in according to the cell line sensitivity severity from up to bottom. The comparisons between each two groups at 40 nm Eto concentration were obtained with unpaired *t*‐test, graphpad prism 7.03: WT + KU55933 (31%) vs WT (91%), *P* < 0.0001 (****); *DNA‐PKcs^∆^* (64%) vs WT (91%), *P* = 0.0108 (*); *DNA‐PKcs^∆^ *+ KU55933 (10%) vs WT + KU55933 (31%), *P* < 0.0001 (****); *DNA‐PKcs^∆^ *+ KU55933 (10%) vs* DNA‐PKcs^∆^* (64%), *P* = 0.0004 (***). *XLF^∆^* (39%) vs WT (91%), *P* = 0.0003 (***); *XLF^∆^ *+ KU55933 (10%) vs WT + KU55933 (31%), *P* < 0.0001 (****); *XLF^∆^ *+ KU55933 (10%) vs* XLF^∆^* (39%), *P* = 0.0006 (***). *XRCC4^∆^* (22%) vs WT (91%), *P* = 0.0001 (***); *XRCC4^∆^ *+ KU55933 (6%) vs WT + KU55933 (31%), *P* < 0.0001 (****); *XRCC4^∆^ *+ KU55933 (6%) vs* XRCC4^∆^* (22%), *P* = 0.0056 (**). *PAXX^∆^* (76%) vs WT (91%), *P* = 0.0898 (n.s.); *PAXX^∆^ *+ KU55933 (33%) vs WT + KU55933 (31%), *P* = 0.7206 (n.s.); *PAXX^∆^ *+ KU55933 (33%) vs* PAXX^∆^* (76%), *P* = 0.0050 (**).

ATM and DNA‐PKcs belong to the same family of protein kinases 33. To determine the genetic interactions between *ATM* and the *XRCC4* paralogues in human cells, we first exposed *DNA‐PKcs^∆^* (Fig. 5A), *PAXX^∆^* (Fig. 5B), *XLF^∆^* (Fig. 5C), and *XRCC4^∆^* (Fig. 5D) HAP1 cells to KU55933 and observed no significant difference in sensitivity to KU55933 between these cell lines and the WT control when the KU55933 concentrations were up to 7.5 μm. Next, we exposed these HAP1 cells to Eto and found that inhibition of ATM resulted in increased sensitivity of *DNA‐PKcs^∆^* (Fig. 6A), *XLF^∆^* (Fig. 6B), and *XRCC4^∆^* (Fig. 6C) HAP1 cells to Eto, while the sensitivity of *PAXX^∆^* HAP1 cells to Eto was at levels comparable to the WT controls (Fig. 6D). We concluded that ATM functions redundantly with DNA‐PKcs, XLF, and XRCC4, but not PAXX, in Eto resistance in human HAP1 cells.

### NHEJ‐knockout HAP1 cells possess increased spontaneous genomic instability

**Figure 7:**
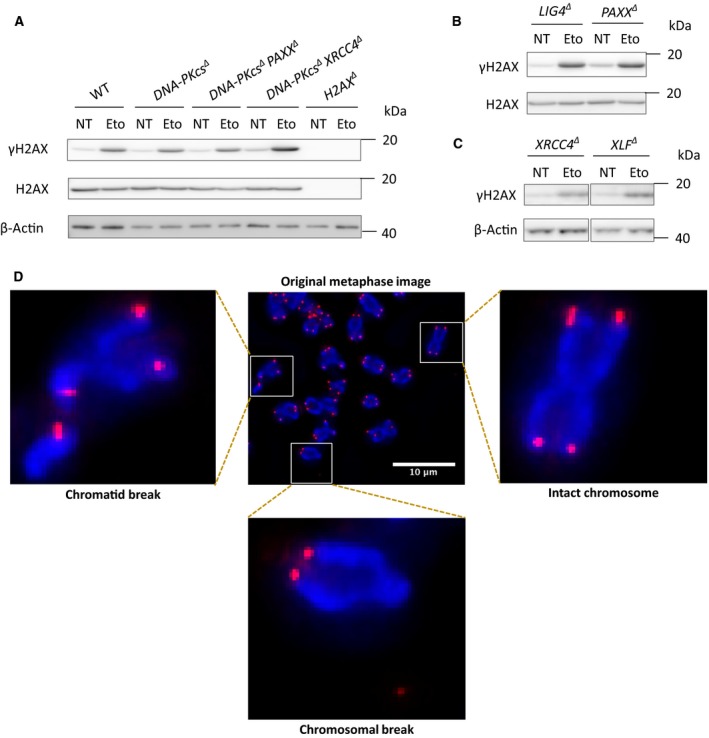
Genomic instability in the HAP1 cells lacking NHEJ factors. WB analyses of γH2AX expression with or without Eto treatment (10 μg·mL^−1^, 2 h) in WT, *DNA‐PKcs^∆^*, *DNA‐PKcs^∆^ PAXX^∆^*, *DNA‐PKcs^∆^ XRCC4^∆^*, and *H2AX^∆^* (A); *LIG4^∆^* and *PAXX^∆^* (B); *XRCC4^∆^* and *XLF^∆^* HAP1 cells (C); β‐actin and H2AX were used as loading controls for WB (Eto). Examples of the intact chromosome and cytogenetic abnormalities, a chromosomal break, and a chromatid break. Red signals indicate telomeres (Cy3), and blue signals correspond to chromosomal DNA (DAPI) (D). The scale bar length in the figure corresponds to 10 μm. The quantification with numbers and percentages of different types of aberrant metaphases of each cell line is shown in Table 2.

To determine the effect of DNA repair factor deficiency on genomic stability in HAP1 cell lines, first, we analyzed the γH2AX presence in the HAP1 cell lines, and we detected the presence of γH2AX in all the nontreated (NT) HAP1 cell lines, except the *H2AX^∆^* cell line, which was the negative control (Fig. 7A–C). Moreover, 10 μg·mL^−1^ Eto exposure for 2 h increased the level of γH2AX expression in all the HAP1 cell lines (Fig. 7A–C). Then, we applied the telomere fluorescence *in situ* hybridization (T‐FISH) assay to quantify the chromosomal and chromatid breaks in metaphases (Fig. 7D and Table 2). We found that both in WT and knockout cells, the majority of cytogenetic abnormalities were chromosomal breaks. Although *H2AX^∆^* cells had a relatively high proportion of chromatid breaks (0.21 breaks per cell) when compared to NHEJ‐knockout cells, the chromosomal breaks (0.62 breaks per cell) in the cells lacking H2AX were even higher (Table 2), strengthening previous observations in *H2ax*‐deficient murine cells 34, 35.

**Table 2:** Telomere FISH analysis of metaphases from HAP1 cells. Overall, 77 to 127 total metaphases of each indicated genotype were analyzed. Numbers and percentages of different types of aberrant metaphases are indicated.

Genotype	Total metaphases	Chromosomal breaks	Chromatid breaks	Breaks/cell (average)	Aberrant metaphases, %
WT	88	13	1	0.16	15
*DNA‐PKcs^∆^*	127	25	10	0.28	17
*PAXX^∆^*	114	18	3	0.18	18
*DNA‐PKcs^∆^ PAXX^∆^*	99	14	8	0.22	19
*XLF^∆^*	124	24	9	0.27	19
*LIG4^∆^*	84	23	7	0.36	27
*XRCC4^∆^*	92	32	9	0.45	33
*DNA‐PKcs^∆^ XRCC4^∆^*	77	28	3	0.40	34
*H2AX^∆^*	86	53	18	0.83	52

The levels of genomic instability in *DNA‐PKcs^∆^ PAXX^∆^* double‐knockout HAP1 cells (0.22 breaks per cell) were in the range of *DNA‐PKcs^∆^* (0.28 breaks per cell) and *PAXX^∆^* (0.18 breaks per cell) single‐knockout HAP1 cells. While *XRCC4^∆^* cells showed considerably higher genomic instability (0.45 breaks per cell), the genomic instability in *DNA‐PKcs^∆^ XRCC4^∆^* double knockouts (0.40 breaks per cell) was similar to that in *XRCC4^∆^* cells. These observations validated our previous conclusion that DNA‐PKcs functions epistatically with PAXX and XRCC4. Finally, *XLF^∆^* cells possessed 0.27, and *LIG4^∆^* 0.36 breaks per metaphase, correspondingly (Table 2). Overall, the inactivation of NHEJ factors in HAP1 cells resulted in increased genomic instability.

## Discussion

Here, we custom‐generated and characterized multiple single‐ and double‐knockout human HAP1 cell lines and systematically analyzed the genetic interactions between *DNA‐PKcs/ATM* and the *XRCC4* paralogue genes during DSB response in HAP1 cell lines. We found that *PAXX^∆^* cells were less sensitive to Eto and had fewer chromosomal breaks per cell when compared with *XLF^∆^* cells, and lack of XRCC4 resulted in the strongest phenotype. The severity order of mutant phenotypes is *PAXX^∆^* < *XLF^∆^* < *DNA‐PKcs^∆^* < *XRCC4^∆^ = LIG4^∆^*.

Inhibition of DNA‐PKcs in *PAXX^∆^* HAP1 cells suggested potential redundant functions between PAXX and DNA‐PKcs (Fig. 3C). However, it was not confirmed in *DNA‐PKcs^∆^ PAXX^∆^* double‐knockout cells, which resembled the phenotype of *DNA‐PKcs^∆^* single‐knockout models (Fig. 4a,B). One could speculate that the difference between inhibition and depletion by genetic inactivation of *DNA‐PKcs* might be due to the presence of a structurally intact protein or residual DNA‐PKcs‐dependent phosphorylation in the first case. Considering that the *DNA‐PKcs^∆^ PAXX^∆^* double‐knockout cells have no DNA‐PKcs protein expressed, we concluded that DNA‐PKcs and PAXX function epistatically in response to Eto‐induced DSBs in human HAP1 cells. These results strengthened conclusions obtained using distinct cellular models and DSB‐inducing reagents 13, 36. Moreover, WT and *PAXX^∆^* cells possessed similar sensitivity to Eto (Fig. 6D) when exposed to ATM inhibitor KU55933, suggesting that PAXX functions epistatically with ATM to promote Eto resistance in human HAP1 cells. This conclusion is in line with the previous observation that PAXX does not functionally overlap with ATM in murine cells 11, 37.

Both DNA‐PKcs and ATM inhibitors (NU7441 and KU55933) significantly increased *XLF^∆^* HAP1 cells' sensitivity to Eto (Figs 3B and 6B), indicating that XLF functions redundantly with both DNA‐PKcs and ATM in DSB response. This conclusion is in line with previous observations that XLF has redundant functions with both DNA‐PKcs and ATM in normal mouse development 9, 32, 38.

*DNA‐PKcs^∆^* HAP1 cells exposed to ATM inhibitor displayed significantly increased sensitivity to Eto (Fig. 6A), suggesting that DNA‐PKcs and ATM function redundantly in Eto resistance of human HAP1 cells. This result highlights previous observations when murine models were used 21, 22, 39, 40.

While inhibition of ATM resulted in increased *XRCC4^∆^* cells' sensitivity to Eto (Fig. 6C), neither inhibition nor depletion of DNA‐PKcs changed *XRCC4^∆^* cells' sensitivity to Eto (Figs 3D and 4C,D). Thus, XRCC4 functions redundantly with ATM, but epistatically with DNA‐PKcs.

In conclusion, we found that DNA‐PKcs functions redundantly with XLF, but not with PAXX and XRCC4, and ATM functions redundantly with DNA‐PKcs, XLF, and XRCC4, but not with PAXX. Our study based on the human HAP1 cell line model validated and strengthened the previous observations of the possible genetic interactions between *Paxx*, *Xlf*, *Atm,* and *Dna‐pkcs* in genetically modified murine cell lines or knockout mouse models 9, 36, 38, 39, 41. Overall, we found that HAP1 is a suitable model to study the genetic interactions in human cells.

## Conflict of interest

The authors declare no conflict of interest.

## Author contributions

MX and VO planned and interpreted experiments, which were performed by MX. MX wrote the paper with the help of VO.
