# Corrigendum to: Genetic interaction between DNA repair factors PAXX, XLF, XRCC4 and DNA‐PKcs in human cells

**DOI:** 10.1002/2211-5463.12777

**Published:** 2020-01-06

In Figure 1 of the article by Xing and Oksenych 1, the western blots against PAXX and β‐actin for all four lanes in Figure 1A and the western blots against XRCC4 and β‐actin in the lanes for WT and XRCC4^∆ ^cells in Figure 1B were reproduced from an earlier publication from the Oksenych laboratory 2 without acknowledgement. This does not affect the conclusions of this paper. A corrected version of Figure 1 and the accompanying legend are provided here:

**Figure 1:**
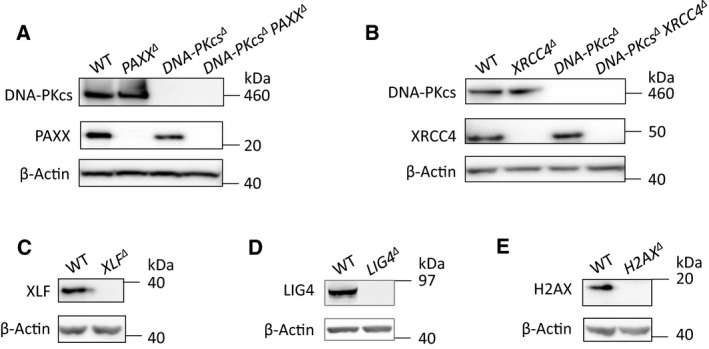
Verification of HAP1 cells by western blot. Western blot (WB) analyses of DNA‐PKcs and PAXX expression in WT, *PAXX^∆^*, *DNA‐PKcs^∆^* and *DNA‐PKcs^∆^ PAXX^∆^* HAP1 cells; (A); expression of DNA‐PKcs and XRCC4 in WT, *XRCC4^∆^*, *DNA‐PKcs^∆^* and *DNA‐PKcs^∆^ XRCC4^∆^* HAP1 cells (B); expression of XLF (C), LIG4 (D) and H2AX (E) in WT, *XLF^∆^*, *LIG4^∆^* and *H2AX^∆^* HAP1 cells; and β‐Actin was used as a loading control for WB. The western blots against PAXX and β‐actin for all four lanes in Figure 1A and the western blots against XRCC4 and β‐actin in the lanes for WT and *XRCC4^∆^* cells in Figure 1B were reproduced from an earlier publication from the Oksenych laboratory 2.
